# What do we know about the influence of vacuum on bacterial biocenosis used in environmental biotechnologies?

**DOI:** 10.1007/s00253-019-10213-6

**Published:** 2019-11-29

**Authors:** Anna Gnida

**Affiliations:** 1 Environmental Biotechnology Department, Faculty of Environmental Engineering and Energy, Silesian University of Technology, Gliwice, Poland; 2 Biotechnology Centre, Silesian University of Technology, Gliwice, Poland

**Keywords:** Vacuum, Pressure, Environmental biotechnology, Bacteria

## Abstract

The article aims to show the increased interest in the applications of vacuum in the area of environmental biotechnology and the lack of research related to the effects of vacuum on bacteria and microbial communities. Information on the impact of vacuum on bacteria is limited and often comes from unrelated research fields. In most cases (astrobiology research, food preservation technologies), the exposure of microorganisms in vacuum is permanent for the whole life of a cell. In environmental science applications, the exposure of microorganisms containing media such as sludge or soil in vacuum is rather persistent, and lower values of vacuum are used. Vacuum is used or proposed to be used in wastewater treatment, anaerobic digestion, sludge treatment, soil remediation and mining. Usually, vacuum is used to remove gases from the test medium, so a purely physical process is applied. However, most reports show the influence of vacuum on biological processes and its efficiency, as well as on the community structure.

## Introduction

Pressure is an important parameter that can profoundly influence cell growth and proliferation. Life on Earth is supported by pressure conditions from 310 hPa (top of Mount Everest) through 1013 hPa at sea level to 1.1^10^6^ hPa at the Mariana Trench, the oceans’ deepest point. This is probably the reason why the effect of pressure on living systems and biomolecules has been intensively studied, mainly regarding pressures above the atmospheric value (atmospheric pressure) and much higher than the highest natural value (the food industry uses pressures up to 8^10^6^ hPa). Air pressure decreases exponentially from the highest value above sea level, and most terrestrial life is supported in atmospheric pressure conditions close to values at sea level. Vacuum is represented by the pressure below Earth sea level. It is commonly used for degassing and transportation of matter. In the food industry, it is used for packing in order to preserve food products from spoiling and prolong their shelf life (e.g. Hernández-Macedo et al. 2011). Research on the influence of vacuum on bacteria and yeasts is one of the most developed fields. Another much studied area is the astronomical science, and it focuses on the probability of life on Mars, where the atmospheric pressure on the surface ranges from 0.3 to 11.5 hPa (e.g. Frösler et al. 2017; Podolich et al. 2017). The number of publications in these two areas would allow for the creation of separate literature reviews.

Biotechnology is a field where the knowledge about features and abilities of microorganisms, mechanisms of bioprocesses and life’s optimal conditions allow to create new or optimise current technologies to be used in order to serve humans. The existing data on the vacuum effect on biological processes and microbial cells allows to assume that the application of vacuum can be much more extended than currently, bringing new possibilities for the improvement of control strategies and the efficiencies of different processes.

**Fig. 1:**
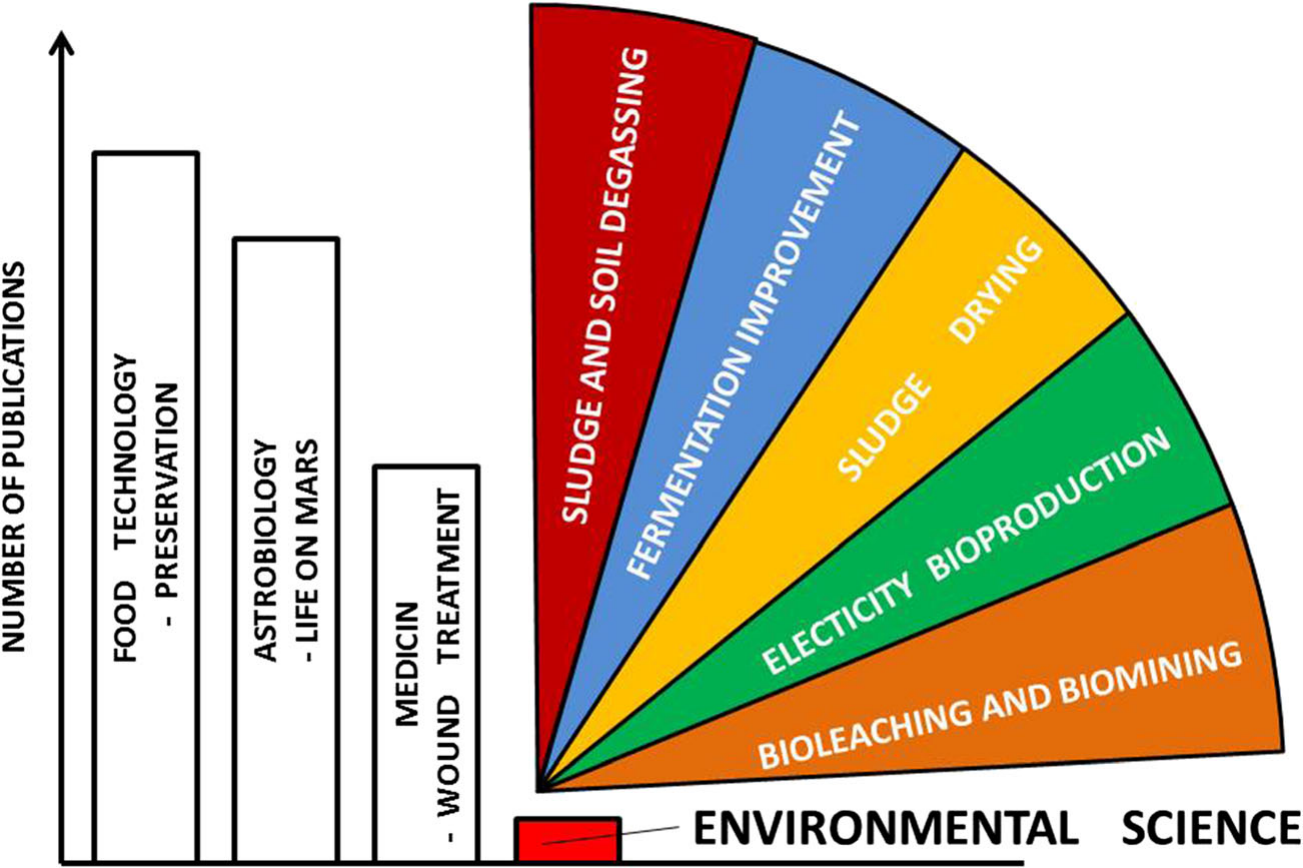
The most common applications of vacuum in environmental biotechnology

Table 1 presents the results of searching publications containing in the title, abstract or keywords vacuum bacteria and selected words representing the mentioned research areas. Assuming that the appearance of the word vacuum and bacteria in the title, abstract or keywords is synonymous with conducting research on the effects of vacuum on bacteria in an adequate research area, it can be seen that vacuum is often used in environmental research; however, the number of publications on bacteria is small. Although this assumption is highly imperfect, it shows the lack of biological research on the impact of vacuum on bacteria. Environmental biotechnology is mainly based on the use of very diverse bacterial biocenosis, and the research is based on studies of the entire biocenosis, its abundance or biodiversity. In the case of biological research in the field of food technology or astrobiology, research is often carried out with the use of pure cultures or mixtures of a few species. In these cases, the word bacterium is not present but the name or names of the strains tested. The number of publications regarding the effect of vacuum on bacteria is therefore difficult to estimate; however, from the author’s subjective observations, it appears that the relationships between the areas discussed are similar to those shown schematically in Fig. 1.

**Table 1:** The number of publications containing in the title, abstract or keywords the word vacuum and other selected words (according to https://www2.scopus.com)

Field of science	Word in title or abstract or keywords	Word in title or abstract or keywords	Number of papers	
			Without “bacteria” in title or abstract or keywords	With “bacteria” in title or abstract or keywords
Food technology	Vacuum	Food preservation	824	341
	Vacuum	Shelf life	1727	595
Medicine	Vacuum	Wound therapy	4428	313
	Vacuum	Wound treatment	4034	303
Astrobiology	Vacuum	Space	24217	126
	Vacuum	Martian conditions	124	14
	Vacuum	Mars	561	25
Biotechnology	Vacuum	Biotechnology	375	37
Environmental biotechnology	Vacuum	Environmental biotechnology	24	6
	Vacuum	Soil bioremediation	52	0
	Vacuum	Biological sludge	84	13
	Vacuum	Biomining	1	0
	Vacuum	Waste fermentation	68	8
	Vacuum	Sludge drying	112	4
	Vacuum	Sludge degassing	14	0
	Vacuum	Wastewater degassing	10	0
	Vacuum	Electricity production	173	1
	Vacuum	Microbial fuel cell	19	4

In many examples presented in the next sections, the use of vacuum is not intended to treat microorganisms but to enforce certain physicochemical processes. In the meantime, however, microorganisms are exposed to vacuum. The most common applications of vacuum in environmental biotechnology are presented in Fig. 1. This short review aims to show the potential of vacuum and the gaps in our knowledge.

## Use of vacuum in environmental biotechnologies

The environmental technologies and the applications of vacuum where it may have the potential to influence exposed bacteria are presented in Table 2. In the aforementioned applications, the goal is to achieve specific desired physical or chemical effects, without deliberately affecting microorganisms. The obtained response of microorganisms is either not recognised or recognised as a consequence of physicochemical but not biological processes.

**Table 2:** Use of vacuum in selected branches of environmental biotechnology

Branch of technology	Aim of lowering pressure	Effect on process or microbials	Pressure, mbar	Exposure	Source
Wastewater treatment	Degassing	Improvement of sludge settling	50	Intermittent, app. 30 sec	Maciejewski et al. 2010
	Microbial fuel cells	Higher production of electricity	50–750	Continuous	Xiao et al. 2013
Sludge treatment	Sludge disintegration	Damage of bacterial cells	20	5–30 min	Abbassi 2003
	Degassing in anaerobic digester	Higher fermentation efficiency, change in community structure	not given	Continuous	Rajhi et al. 2016
	Sludge drying	Improvement of drying rates	74–268	Continuous	Sagberg 2004; Yan et al. 2009
Soil treatment	Soil remediation, suction of gases	Volatile compounds removal	not given	Intermittent, unspecified	Thornton et al. 2007
	Bioleaching, intermittent liquid removal	Change in community structure	not given	Intermittent, 90 sec	Rzhepishevska et al. 2005
	Biomining of peatland	Change in community structure	not given	Intermittent, unspecified	Croft et al. 2001

## Wastewater treatment

In wastewater and sludge treatment, vacuum is usually used for degassing of wastewater or sludge. The degassing process is based on Henry’s law, and in its very general form, it deals with the dependence of gas solubility on the pressure value. The degassing process removes gas bubbles from mixed liquor (activated sludge) and reduces the dissolved gases concentration. In the sludge that reaches the final sedimentation stage (atmospheric pressure), the dissolved nitrogen gas concentration is well below the saturation level. Thus, gas molecules produced in the sedimentation tank dissolve in the liquid instead of forming bubbles. Accumulated bubbles can result in sludge flotation what is undesirable. As a consequence of degassing, the activated sludge settles well at the settling stage without the formation of a layer of partially settled or floating solids (Maciejewski et al. 2010; Gnida and Witecy 2018). The separation of solids (activated sludge) from treated wastewater is one of the most crucial and problematic issues of a wastewater treatment plant (WWTP). The enhanced ability of activated sludge to settle and thicken at the final sedimentation stage allows WWTP operators to increase the sludge amount in the biological reactor (i.e. amount of bacteria involved in the process) and improve the removal of contaminants. The degassing of activated sludge occurs due to a short-term (30 sec) reduction of pressure to ca. 50 hPa. As the degassing unit is located between the reaction stage and the settling stage, activated sludge is treated by vacuum once in a time equal the hydraulic retention time. The system has been implemented in several places in Poland, Sweden, China, Estonia and Canada, and it presents a great improvement in regards to nutrients removal, especially denitrification (Maciejewski and Timpany 2008; Maciejewski et al. 2010; Maciejewski et al. 2013). Is the effect, however, simply the result of an increase of bacterial/sludge density in the reaction tank? In fact, only the settling properties of activated sludge and the overall WWTP control results have been presented so far (Maciejewski and Timpany 2008; Maciejewski et al. 2010; Maciejewski et al. 2013; Haghighatafshar et al. 2017). However, there is a suspicion that sudden vacuum conditions can be a stressful factor for bacteria and cause a change in their metabolic activity (Gnida 2015; Gnida 2018).

## Anaerobic digestion

In anaerobic treatment, vacuum could be useful in controlling the anaerobic digestion process. From an industrial point of view, the optimisation of hydrogen production is of the highest importance. In order to reach a high hydrogen production, the produced hydrogen has to be removed from a headspace as the accumulation of hydrogen causes inhibition of hydrogenesis (Rajhi et al. 2016). This is commonly achieved by stripping with an inert gas (e.g. N_2_ gas). The idea of using vacuum for this purpose has been verified by several research teams, which obtained opposite results. Sonnleitner et al. (2012) argue that the application of pressure of 305 hPa allows nitrogen stripping to be omitted and hydrogen yield was close to the theoretical maximum. In contrast, continuous reactors vacuumed to 284 hPa by Kataoka et al. (1997) and 600 hPa by Clark et al. (2012) showed no or a non-significant effect on gas production, respectively. Meanwhile, other researchers found that vacuum-derived partial pressure may generate different hydrogen production yields depending on the value of hydraulic retention time. All vacuumed reactors, however, revealed a high hydrogen production efficiency with the desired product composition reached in acetate and butyrate (Lee et al. 2012). The effect of applying vacuum on dark fermentation systems was analysed by Rajhi et al. (2016). The dark fermentation is fermentative conversion of organic substances to biohydrogen in the absence of light. The application of vacuum promoted an increase in the diversity of hydrogen-producing bacteria (*Clostridium*), as well as favoured the dominance of acetoclastic over hydrogenotrophic methanogens. In addition, significantly more OTUs (operational taxonomic units) were found in the vacuum-exposed community. The application of vacuum (its value is not presented, but the used equipment suggests that it was not very low) caused a higher biogenic hydrogen and methane production, which is a very promising perspective. Unfortunately, there are no other data available regarding the influence of vacuumed headspace on a fermentative bacterial community. Both positive and contradictory results, however, testify to the research and development potential of the application of vacuum in anaerobic processes.

One of the ways to improve anaerobic sludge digestion and accelerate rate-limiting hydrolysis is sludge disintegration. There are plenty of methods of a mechanical, chemical or biological nature that are used for that purpose and are described by Zhen et al. (2017). Although this review is reliable, it does not include the technology based on the use of vacuum presented by Abbassi (2003). Sludge exposed to 20 hPa for 5–30 minutes was investigated. Vacuum was found to interrupt bacterial cells and cause the release of cell content, thus increasing the value of chemical oxygen demand in the supernatant. Such treatment enhanced hydrolysis of bacterial cells and improved sludge digestion. Another example of using vacuum to reduce waste and increase the energy recovery is to combine it with the steam treatment. This was found to promote the digestive efficiency of the sludge (Itoh et al. 2017).

The drying of sewage sludge has been found to be significantly more effective when assisted by vacuum (Sagberg 2004; Zhen et al. 2017). Vacuum conditions allow use of lower temperatures in the process (i.e. 50–90^o^C). Unless a lower temperature is used, the vacuum treatment decreased the number of *Escherichia coli* and spores of sulphite-reducing anaerobic bacteria (*Clostridia*) and f-specific bacteriophages in comparison to non-vacuumed samples (Sagberg 2004).

## Soil treatment

Vacuum is also used in the treatment of soil and groundwater environments for soil dewatering and reduction of pore water pressure, as well as the removal of volatile compounds from soil air. A vacuum heap biostimulation system was used for the treatment of excavated soil, which is recommended when the timeframe for its treatment is short. The improvement in the removal of polycyclic aromatic hydrocarbons (PAHs) after applying vacuum was significant, but no microbiological analysis was performed, and, therefore, there is no information regarding the vacuum extent (Eiermann and Bolliger 1995). Similarly, other researchers (Just and Stockwell 1993; Thornton et al. 2007) argue that the use of vacuum improves the efficiency of solvent-contaminated soil treatment and enhances further bioremediation. No microbiological research has been performed.

Intermittent vacuum conditions were used for the bioleaching of sulphidic tailings. Here, an extended community analysis was performed showing that a novel vacuum-based bioreactor provided conditions that were useful for bioleaching for selected bacterial populations. Setting aside numerous differences in configurations and chemical and physical conditions between a vacuum bioreactor and a reference (stirred) reactor, the molecular analysis of the bacterial community showed slight differences in the two assemblages (Rzhepishevska et al. 2005). It has not yet been assessed, however, whether the vacuum value and its duration influence the leaching efficiency and the bacterial activity. The mixture of solid matter (ore, tailings, etc.) and bacteria was exposed to alternating vacuum conditions where one cycle takes ca. 90 seconds and the vacuum condition comprises about 30% of the cycle. Thus, the bacteria are intermittently under a pressure stress that is not found in natural conditions. The authors do not provide information about the level of vacuum.

There are also examples of soil biotechnologies where microorganisms are exposed to vacuum. In soil bioremediation, vacuum is used to remove groundwater or soil vapour for further treatment in dedicated instillations. The influence of such treatment to indigenous soil bacteria is rather limited, as most attention is focused on the efficiency of the removal of contaminants. Such treatment, however, can change the microclimate and humidity of the ground. Canadian researchers found that vacuum extraction in peatland while mined disturbs the bacterial population and the bacterial biomass carbon (Croft et al. 2001). Significantly fewer total bacteria were found in vacuum-treated land, especially of hemicellulolytic and cellulolytic properties. At the same time, there were more *Actinomycetes*.

## Microbial fuel cells

Very promising news comes from research concerned with microbial fuel cells. The introduction of vacuum into microbial fuel cells caused only slight changes in the community structure, but the metabolic activity changed significantly. The attachment between extracellular polymeric substances and one of the electrodes was much stronger, resulting in the generation of power that was seven times higher as compared with the atmospheric environment (Xiao et al. 2013). Unfortunately, there is only one such report.

## Molecular biology analysis

Apart from environmental biotechnology applications, there are some data about the influence of vacuum on bacterial cells. Hypobarophilic bacteria (Schuerger and Nicholson 2016) or yeasts (Gamage and Ohga 2017) were recovered from soils at high altitudes or from permafrost. The yeasts grew better in lower pressures of 850 hPa (Gamage and Ohga 2017) showing higher mycelial growth. Nicholson et al. (2010) found that the putative low-pressure barrier for the growth of Earth bacteria is ca. 25 hPa. The same authors argued that at the near-inhibitory low pressure of 50 hPa, *Bacillus subtilis* evolved an enhanced growth ability. A pressure downshift caused an up-regulation of some genes, and the regulation was different in response to the temperature versus the pressure downshift (Fajardo-Cavazos et al. 2012). The latest reports provide data that permafrost bacteria from the genera *Bacillus*, *Carnobacterium*, *Clostridium*, *Cryobacterium*, *Exiguobacterium*, *Paenibacillus*, *Rhodococcus*, *Serratia*, *Streptomyces* and *Trichococcus* may grow even at 7 hPa (Schuerger and Nicholson 2016).

Sarapirom et al. (2011) investigated changes in the topological form of extracellular plasmid DNA (deoxyribonucleic acid) due to lesions in DNA under vacuum conditions. The results show that vacuum can cause an increase in the relaxed form by about 50% as compared with that of the natural control and that this mainly occurs when the pressure rapidly changes. This result indicates that the DNA change is predominantly caused by the pressure change instead of the pressure itself, even though the pressure is very low.

Such information, originating from the astrobiological field, may hint at what happened on a molecular level with sludge bacteria, biofilm or other microorganisms when exposed to constant or periodical vacuum. How does this affect the community structure and ecology?

## Conclusion

Studies on the influence of pressure on microorganisms are quite extensive, while the influence of vacuum is a rather little recognised field. Here, only such applications of vacuum among environmental biotechnologies are presented where the bacteria involved in the technology are exposed to vacuum conditions. The few research reports on the impact of vacuum on biological processes come from various areas of environmental biotechnology. Most of the obtained results indicate that vacuum can also have biological, besides physicochemical, effects on microorganisms or communities. The results of molecular studies indicate that the effects obtained will depend not only on the value and duration of exposure but also on the degree of change in the value and the cyclicality of action. In my opinion, there is an open research field here, awaiting to be explored, which holds a lot of interest.
